# Supplementary material for: Laccase-mediated biotransformation potential for fluorinated compounds by geographically diverse human gut microbiota
Source: mBio. 2026 Aug 31;17(10):e01026-26. doi: 10.1128/mbio.01026-26 (PMC13712835; doi:10.1128/mbio.01026-26)
Supplement: Supplementary file 1 [file mbio.01026-26-s0001.docx]

# Supplementary figures

**Figure S1:** Completeness and contamination of recovered MAGs as evaluated by BUSCO.

**Figure S2:** Per-sample abundance measured as reads per million (RPM) of contigs containing putative *rpoB* genes plotted against URCA index values. A lower URCA index value indicates a greater degree of urbanization.

**Figure S3:** Percentage of strains within the BacDive database isolated from different sources for species where laccase-coding contigs were found. Strain isolation sources were categorized as animal, food, human gut, human non-gut or other.

**Figure S4:** Taxonomic classification of MAGs which contained contigs with laccase-coding genes.

**Figure S5:** SDS-PAGE of each laccase his-column purification. Wash represents fractions from the His-column before sample elution with imidazole. Elute represents fractions after his-column elution and buffer exchange column. Vei1_Δ120, Vei2_Δ101, Lac, Kur, and Ent2 all showed reasonable purity and yield. Ent1 did not show a discernable band at the predicted size. Akk1 and 2 both showed induction by IPTG (pre- and post-induction fractions labeled accordingly), but no clear band was discernable in the final elution fractions among the significant impurities.

**Figure S6:** Alphafold 3 predictions of laccase structures. For Vei1 and Vei2, the truncated portions in Vei1_Δ120 and Vei1_Δ101, respectively, are shown in red.

**Figure S7**: Chemical structures of the 11 selected fluorinated compounds used in this study.

# Supplementary tables

**Table S1**: Overview of laccases tested in this study. Seq = Protein sequence. Abx = Antibiotic. Codon-optimized sequences are at https://github.com/MSM-group/gut-laccases/tree/main/sequences.

| **Name** | **Seq** | **Vector** | **Abx** | **Cut sites** | **Supplier** | **Scientific name** |
| --- | --- | --- | --- | --- | --- | --- |
| Vei1 | MKKYSNYMKALLVAVAITGGTAIVSAHDTVTTNDCCYDSATATNSQNMTANMSSMMGGMVNNMMNNQSSANNSSMHSMMNGQNSMDSNSMHSMMSGMMNGGMMGMTATNSMNGMMGMMGHMHGVDDVPALKQVQQAAKPLAIPPMQKGTVDENGVRHLEVTAQEGKTAIKDGALTDTYGYNGSVLGTTLYMKHGEKVAIKLKNELPEKTTYHWHGMVVRSDVDGGPHYPIAANGGTGEVEFTVDQSANTAWYHPHAMGLTASQVYKGLAGFIYVDDNKEGALNLPHTYGVDDIPVAIQERNFTADNQWDYEKDYNADGVYGDTLVVNGTINPYFDVKTNMVRLRLLNGSNARTYTLQLSDMSPMMQVAGDGGILPWPVPKQSITIAPGERVEVLVDFSKYKDALPSLVTTNGTNGTANVLNFKRGADTLTAEAKVNTDMAAWNYNNMNASDDLTKPADKSIVMSGMAQNVMINGKKFDMGRIDFTSKLGSTEIWDVSNADPGMMGMMHPFHVHGVQFEVLSRNGRPVDAAEKGLKDTIQVDPGEHVRIRLHFTKPGVFMVHCHILEHEENGMMLQLEVK | pRSETA (full sequence)  pET28a+ (Δ120) | Amp (full sequence)  Kan (Δ120) | EcoRI/HindIII (full sequence)  Nde1/Xho1 (Δ120) | IDT  TWIST | *Veillonella* |
| Vei2 | MKINQMKLAKLALAVTLMTGSGYVVNAQMMNGMMNQSMMSQDVMDQDSMNSMMNTMMGQSSSNQNYGNGMMNGSMMNGMMNQNSMHNTMMNNMMSGQGMMGMHDEAMTLHQSTQAPRPLAIPPLVKGTPDGNGRMVYNVTAQTGESDIKDGQKTATYGYNGNVLGPALLMKRGEKVTINLTNNLPEATTFHWHGLVVRSDVDGGPHYPIAANGGTGKIEFTVNQPAGTAWFHPHAMGSTASQVYKGLAGLIVIDDNEIGKLNLPHEYGVDDIPVAIQDRLFTKDNQWDYKSAYSDDGVYGDTLVVNGTINPYFEVPKKLVRVRLLDGSNARNMTLRLSDGSTMYQIASDGGLLEAPVATQEITLVPGERAEVLIDFSRYANADKLPSLVTQNGTINVLEFRKAASWNTDSAASVNNTIPTWNFETKKHDEELQKAATTDKIAQHIVMSGMAKDVTINGKKFSMDRIDLTAKLGSTEVWEISNSDGMMGMIHPFHIHGVQFEVVSRNGVPTTPDERGLKDTISVAPGENVRIRIHFTQPGIFMVHCHILEHEENGMMLQLQVK | pCDF-Duet-1 (Δ101) | Spec (Δ101) | BsaI/BsmBI (Δ101) | Joint Genome Institute | *Veillonella* (Δ101) |
| Ent1 | MKNIRTSFSDSPSKETMQEERPNRNTMGNMMTNEQNIELKNISQSEQPLLIPPLLEPGSEDDENISYNITAKEGEFQIKEGKKTKTLGYNRDFLGPVIRMKQGQKVTLTTTNQLKSKTSFHWHGLKIPSEVDGGPHQVVEPGEKKVISFTVDQDASTLWFHPHPEGETASQVYRGLAGLMYIDDENSDSLNLPNNYGKDDIPLIVQDKSFDSDNQIDYETDFYSDGTQGDTLLVNGTINPYIEVNSRWMRYRIISGSNSRNFTFSLKDNQPFYQIASDGGLLNSSTKLTSLTLSPGERAEILVDTNDYKNGDTLKLLANNTVALTMKINKNKSNTTFEPSKKLNSIKDIDTENLDKLDRQKIDLKGMSHMVSINNKQFDADRIDLYKRVNTQEVWEVNNISGMMGGMIHPFHIHGVQFQIISRNGQLPQANERGWKDTVLVNPEETVELLVEFDHEGVFMYHCHILEHEEYGMMGQMEIK | pCDF-Duet-1 | Spec | BsaI/BsmBI | Joint Genome Institute | *Enterococcus dispar* ATCC 51266 |
| Ent2 | MQQNKEEHAEVVFQEEQRQISSRMSNMMGDNQEADLKVVHQTERQLLIPPVLEPSSKSKENVIYDIVAQNGEVQIMDGEKTETLGYNGDFLGPVIRLKKGQKVTINTTNNLDASTSFHWHGLKVASDADGGPHQIIEAGQKKSVTFEVDQEASTLWFHPHSEGETASQVYKGLAGLMYIDDDNSKSLDLPSKYGVDDIPLIVQDKSFSSTNQINYENDFNSDGTKGETLLTNGTINPYIEIKNRWMRYRIVNGSNSRNFTFNLDNDESFYQIATDGGFLNTSVKLSKLLLAPGERAEILVDTQNYKKGKVIHLLANNLVALTMRIENTIDNKEFNPSDSLNTISTLDEKKLEDLTRQSINLSGMSHMVNINNKQFDMERIDLYKKLGTQEIWEVNNISSMMGGMIHPFHIHGVQFQILSRDGNQPALNEQGWKDTVLVNPDEIVELLVKFDREGIFMYHCHILEHEEYGMMGQMEIK | pCDF-Duet-1 | Spec | BsaI/BsmBI | Joint Genome Institute | *Enterococcus durans* ATCC 6056 |
| Kur | MKKTLIASLLVGTALLAACGNEDGATDHTTGASEHQHSMANTHQNGSMDHEQLVLKDNKGTNTLIFPAILKPDSEKGKNVEYSLTAQAGTTELFNGYETKTYGYNGNLLGPTLRLKEGQHVTIHLKNDLPEATTFHWHGLEVSGKADGGPHALIEPGEEKTIQFTVTQQAATLWYHPHPMGNTAQQVYKGLAGLLYIEDDNVEALNLPNDYGKNDFPIILQDRAFVEGKQLDYSKVANSDGTYGDTMMINGVINPVLKTDKKLVRLRILNGSNARNYNVHFDNDMAFTQIATDGGFLNKPQKMKELLLGAGERAEILVDLSKVKGKRVSLVNDQNVVLLPIDVSDTKSAVNASTKLNTIKVDKALLEQQPTKVVKMEGMRENVTINGKKFDEKRVDFTQKKGETEVWEIDNAITDEGGMIHPFHIHGTQFLVLSVNGEKPEPSLQGYKDTITLQPGQKARIAVKFPYEGMYMFHCHILEHEDNGMMGQIEVK* | pCDF-Duet-1 | Spec | BamHI/KpnI | GenScript | *Kurthia* |
| Lac | MAKKVYTDYFFDEPAYNTHDGGYIPLVTPKVEPQPLAIPPLLKPDRQTDTDDYYTVTAQESETQFLPGKKTKTWGYNAGFLGQTIVFRNGKQTHIDLENKLPELTTFHWHGLNVPGPITDGGCHAPVYPGETNHIDFKVHQPAATTWLHAHPCPSTATQVWKGLATMVIIKDDVEDQLPLPRNYGVDDIPLVLQDREFHDDNQFDYRADYDPDGVQGHTALVNGTVNPYFDVTTQRVRLRILDGSNRREWRLHFNDDLEFAQVASDGGILPAPVYMTKVMMTCAERDEIVVDFGQYQPGDEVTLMTDDTPLCRFRIKSFVPDDTKLPEHLVDIPDETPTPDLPVRTITMDGMDDEVALDGKKFDMSRIDARQKVGDVAIWEIRNTNSTENGMVHPFHVHGTQFRVLARNDGPVYPNEHGLKDTVGVNPGETVRIKVKFELTGVYMYHCHIIEHEDGGMMAQIESYDPQHPQTYHLMDMDTLRNAFAKEQGIKPEDVWMPGM | pCDF-Duet-1 | Spec | BamHI/KpnI | GenScript | *Lactiplantibacillus* |
| Akk1 | METVEYDLYVRNTPVNFTGTARPAMSINGSIPGPVLHFTEGDTAVIRVHNMMDTETSFHWHGLLVPNDQDGVPYLTSAPVKPHTTHTYTFPIIQNGTYWYHSHSGLQEQSGLYGAFVVHKRRNDPARRAEDTLPEYTLVLSDWTNENPNEVNRKLHTGSDWFSIRKGSVQSYWEALRAGYVGTKLTSEWKRMNPMDVSDVYYERFLLNGSPHASLPRLKAGDRVRLRIVNGASSTYFWLRYSGGKIRVVASDGKDVVPVDVDRMIIAVSETYDVILTVPETGRAFEFQATAEDTTGSSSLWLGHGEKQPLRPFPKLNYFKNMKRMNGMMTMGGSMKMMTMSSGSMPGMKHMNHNMSGGMDSPRAGGMHMMSMSSGSSHGKHHAEDMQEDEGEVTLTYDMLRSLAKTNLPSGVPVKELHFQLSGNMNRYVWSINGRTLSETDRIMIREGQNVRIILTNNTMMRHPMHLHGHFFRLVNKQGDFSPLKFTVDIQPMATQVIEFNAAEKTRGNWFFHCHILYHMMSGMGRIFTYEDSPPNPQLPHPRQALQHVYAMDRKWYLTVNNDFASNGNIGDLEFGGTRWSIQGEWQTGYKDTRGYEAEARLGRYIGEKQWLYPYIGMDWTYRKGEAGERNMFRQTTRKDRELDGTLGTRYTLPLLLVADARIDTDGKVRLQLERDDIPLTSRLRLSFSLNTDRDYSVGLHYILTPHVSISTNYDNNLHWGVGLMLTY | pCDF-Duet-1 | Spec | BamHI/KpnI | Genscript | *Akkermansia* |
| Akk2 | MKTVEYDLYVRNAPVNFTGVTRQAMTINGNIPGPTLHFTEGDTAVIRVHNLMDTQTSFHWHGLLVPNDQDGVPYLTSAPVKPHTTHTYTFPIIQNGTYWYHSHSGFQEQSGLYGAFIVRKRPDDPARRTEDALPEYTLVLSDWTNENPQEVNRMLHTGSDWFSIRKGSVQSYWEALRSGCMATKLLSEWKRMNAMDVSDVYYECFLLNGIPRASLPRLRGGDRIRLRIVNGASSTYFWLRYAGGKIRVVASDGKDVVPVDVDRMIIAVSETYDVILEVPESGKAFEFQATAEDTSGSSSLWLGHGERQPLRPFPKLNYFKKMKQMNGMMTMGGNMKMHHHGMPTAHSGNMGIMDMKSGSSHGGGHGNMQEDGEGTTLTYDMLKSPSRTTLPSGVPVKELHFMLTGNMNRYVWSINGKTLSETDRIMIREGQNVRIILTNNTMMRHPMHLHGHFFRLVNRHGDFSPLKFTADIQPMDT | pClOX | Kan | BamHI/HindIII | IDT | *Akkermansia* |
| MCO1 | MKKTDNDTTQDSTPDSPSNESRRAFLKTTGATVIAAPAILTSRKSMAQVVPGVAPSPAT  PWQVDLPDAIEPLQPTDLFTDAYRIPQGTANTAYGECGRNRHQRWNDFFGNPQLQPGQADTYELRVKEDNNYSFHPDYPDQLAWTYEGVDGNGQPYHNPTFFARYGRPVIVRLHNELPKDHEGFGTPEISMHLHNLHTPSESDGFPGDYFSPYKAGPTLTKPGNFKDQFYPNIYAGLDEFPKDVDNPVGGDKREALGTLWYHDHTLDFTAANASRGMAGFYLLFDDLDSGDENDPNPAALRLPSYPYDYPLLFQDKRFDASGIHIYDQIDPEGTLGDKITVNGKIEPVLKVAKRKYRLRLLNAGPSRYYEFYLVNAAGTPQTFDYIANDGNLLPTTLRNQTRVRLGVAERGDIVVNFNRYALGTELYLVNRLVQTSTRGPGAVQAPGDRVLKIVVNRTAADNSQVPNTLRPIRRPTAEEIASAPVRRWTFARTSGLWTINSKLVNVKSPAARISKESMEIWDISNPNGGWSHPVHIHFEEGHILQRFVNGAAVAVPAHERGRKDVYNLGPNERIRLFIRFRDFPGKYVMHCHNMIHEDHAMMVRWDIVE | pRSET A | Amp | EcoRI/HindIII | Thermo Fisher Scientific | *Nitrosomonas* |
| mMCO | MTTAAAAGAPLILTSRKSGAQVVVPPSPPTTPWVQELPRAITPLAAVPSLSPAPTLTANTLGGECGRAEHQRFAELTAPGVCGPPELYEMFARENPGWVFNPAYPPQPVWGFSGSAADPATSPGPTLFGHYGHSVICRIHNQLPANHVGFGTPEISTHLHNLHTPSESDGFPGDFYSATIAGPTLGAPGQFQDHFYPMVYAGLDTYGGIGDSREALGTLWYHDHTMDFTAPNLVRGMAGFYLLFDDLDTGNEQTGLRLPSHPYDYPIFLQDKRFDANGILTYDQFDPEGTLGDKVTVNGKIEPVLRVERRKYRFRFLNGGPSRFYEVYLQNRGATSVYTYTYIANDGNLLPAPLLNQFRFRLGVAERAEIVVDFARFPLGTELYFVNQLLQDNTRGPGNVRAPGTRLLKIIVDRNPAQPDTSRVPSALRPLPPIPTDFSGIPVRRWVFERSGGMWSVNGQFFNVNVPRATIAKGAGEIWELVNPENGWEHPVHIHFEEGRIIEKRVNGAVVPIPLHERGRKDVYTLGKDVSFKVFLRFRDFKGKYVMHCHNLIHEDHAMMVRWDIV | pRSET A | Amp | EcoRI/HindIII | IDT | *Methylmirabilota* |
| aMCO | MKKTDNDTTQDSTPDSPSNESRRAFLKTTGATVIAAPAILTSRKSMAQVVPGVAPSPATTPWQVDLPDAIEPLQPTDLFTDAYRIPQGTANTAYGECGRNRHQRWNDFFGNPQLQPGQADTYELRVKEDNNYSFHPDYPDQLAWTYEGVDGNGQPYHNPTFFARYGRPVIVRLHNELPKDHEGFGTPEISMHLHNLHTPSESDGFPGDYFSPYKAGPTLTKPGNFKDQFYPNIYAGLDEFPKDVDNPVGGDKREALGTLWYHDHTLDFTAANASRGMAGFYLLFDDLDSGDENDPNPAALRLPSYPYDYPLLFQDKRFDASGIHIYDQIDPEGTLGDKITVNGKIEPVLKVAKRKYRLRLLNAGPSRYYEFYLVNAAGTPQTFDYIANDGNLLPTTLRNQTRVRLGVAERGDIVVNFNRYALGTELYLVNRLVQTSTRGPGAVQAPGDRVLKIVVNRTAADNSQVPNTLRPIRRPTAEEIASAPVRRWTFARTSGLWTINSKLVNVKSPAARISKESMEIWDISNPNGGWSHPVHIHFEEGHILQRFVNGAAVAVPAHERGRKDVYNLGPNERIRLFIRFRDFPGKYVMHCHNMIHEDHAMMVRWDIVE | pRSET A | Amp | EcoRI/HindIII | IDT | *Thermoproteota* |
